# Multi-Scale Robotics: A Numerical Investigation on Mobile Micro-Tweezers for Micro-Manipulation with Extreme Requirements

**DOI:** 10.3390/mi16010040

**Published:** 2024-12-30

**Authors:** Ahmet Fatih Tabak

**Affiliations:** Mechatronics Engineering Department, Istanbul Ticaret University, 34854 Maltepe, Turkey; aftabak@ticaret.edu.tr

**Keywords:** micro-manipulation, micro-tweezers, adaptive motion control, multi-scale robotics, forced vortex, magnetic field, permanent magnets, dynamic modeling, numerical simulation

## Abstract

An automated micro-tweezers system with a flexible workspace would benefit the intelligent sorting of live cells. Such micro-tweezers could employ a forced vortex strong enough to capture a single cell. Furthermore, addressable control of the position to the vortex would constitute a robotic system. In this study, a spherical micro-object composed of super paramagnetic particles tightly packed in a non-magnetic resin is rotated with a combined magnetic field of permanent magnets. The said magnetic field is articulated by an open-kinematic chain controlled with a simple adaptive PI-control scheme. A vortex is formed as the spherical particle, assumed to be submerged under the surface of fluid, and follows the position and orientation of the external magnetic field. This forced vortex induces a radial pressure gradient that captures the live cell orbiting around the spherical object combined with the inertial effects. Here, a comprehensive mathematical model is presented to reflect on the dynamics of such micro-tweezer systems. Numerical results demonstrate that it is theoretically possible to capture and tow a bacterium cell while meeting extreme tracking references for motion control. Magnetic and fluid forces on the spherical particle traverse the vortex and the bacterium cell, with orbiting and sporadic collusion of the bacterium cell around the spherical particle, and the positions of the end-effector, i.e., the magnets, are analyzed.

## 1. Introduction

The manipulation of micro particles and live cells benefits from robotics [1,2,3]. Bio-mechatronic systems might employ micro-tweezers operating on various physical phenomena dictated by the operational constraints. These include optical micro tweezers [4,5,6], acoustic micro-tweezers [7], hydrodynamic micro tweezers [8,9], micro-pipettes [10], and piezoelectric-based or shape-memory-alloy (SMA)-based micro robotic gripper solutions [11,12]. All of these approaches mean the direct application of stress, whether by means of fluidics, thermal, or acoustics on the captured particle for control and manipulation purposes.

Handling biological material, e.g., live cells, is a delicate task, as structural integrity should be protected even when their mechanical properties are measured [13]. Direct-contact methods might exert constant stress and might result in deformation even, not in trauma. One alternative approach is to eliminate deliberate tactile contact along with the possibility of heat absorption. For instance, employing rotating magnetic fields to generate vortex flows and inducing viscous forces on the cell to select and transport without tethers could be feasible [14]. Furthermore, such an approach would make use of magnetic fields to ensure non-invasive manipulation of the cells.

An electromagnetic field is a minimally invasive system for biological tissues when applied only for a short time and strong enough for the defined task. Moreover, magnetically actuated micro-robots became an integral part of robotics research [15,16,17]. The wide use of magnetic fields in medicine made it a popular choice of actuation in micro-robotic demonstrations [18,19]. Similarly, magnetic fields might be employed for in vitro cell sorting and manipulation purposes. A magnetic particle rotating under the influence of an external field would create circulation in the vicinity. In the case in which the particle is symmetric along the axis of rotation and the viscous forces dominate the inertial forces, the circulation could be modeled as a potential flow [20]. The usefulness of such non-contact micro-manipulation has already been demonstrated with the help of harvesting protein crystals [21]. It is also known that moving the vortex would help transport the particles captured by the circulating flow field [9,22]. However, the theoretical background for the interaction between a rotating particle and a second one captured by the resultant vortex in motion articulated by a robotic system is yet to be well understood in the context of robotics. However, extensive computational fluid dynamics (CFDs) analysis has been carried out for a stream of eddies induced by stationary structures therefore helping with the transportation of particles on micro-scales [23]. This phenomenon is important to synthesize addressable gaits for sorting or pick-and-place applications [24].

Here, with this study, a holistic mathematical model is presented for the closed-loop control of a system multi-scale robotic micro-tweezers. The main goal is to predict time-dependent forces to calculate the traverse of the vortex in the lab frame under the influence of the applied magnetic field. This provides a flexible workspace in which it would be possible to avoid obstacles by following nonlinear gaits. The vortex core, i.e., the spherical magnetic particle, will try to align itself with the rotation of the magnetic field forcing the surrounding fluid to circulate thereby dragging any particle within the liquid. Once a particle is trapped in an orbit, it can be towed alongside. However, they might collide along the way, a phenomenon observed numerically. The vortex is dragged by a three-degrees-of-freedom robotic arm, while the particle itself is a representation of a real bacterium cell, i.e., *E. coli* minicell [25], under the influence of the vortex along with spatial Brownian jumps due to its size. The control algorithm takes measurements from the robotic arm, as well as the bacterium cell itself, to summon a weighted adaptive integral control along with weighted proportional control; therefore, a PI-control scheme further coupling the robotic arm and the bacterium cell constitutes a multi-scale robotic system. The mathematical model is then reconstructed in the MATLAB/SIMULINK environment and solved using a variable-step ordinary differential equation (ODE) solver to demonstrate how a stationary obstacle in the workspace would be avoided. Detailed results on the rigid-body motion of the end-effector of the robotic arm, the spherical magnetic particle, i.e., the vortex core, and the bacterium cell are presented to showcase how the overall system would perform under extreme velocity reference values that would result induce secondary effects.

## 2. Mathematical Modeling

**Figure 1:**
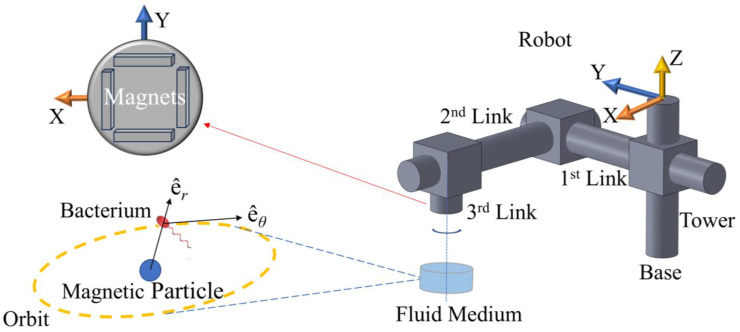
The three-degrees-of-freedom robotic arm (**right**) and magnets at the 3rd Link, i.e., the end-effector, hovering above the fluid medium that contains the hydrodynamic tweezers (**left**). A representative orbit of the bacterium cell around the magnetic particle is denoted by the dashed-circle in orange. The illustrated objects are not equally scaled.

**Figure 2:**
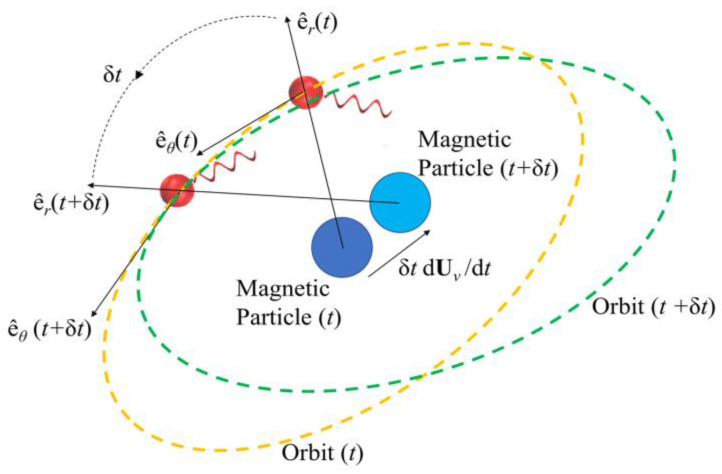
Hydrodynamic tweezers with the core displacing along the XY-plane in an arbitrary direction, thereby dragging and transforming the orbit of the bacterium instantaneously. Unit vectors of the cylindrical coordinates of the vortex core are denoted by ${\hat{e}}_{r}$ and ${\hat{e}}_{\theta }$ in the radial and azimuth directions, respectively.

The physical setup to be modeled is comprised of a robotic arm, four magnets, micro-tweezers, and a bacterium cell. Figure 1 depicts this system as simulated in this study. The hydrodynamic tweezer system is comprised of a spherical magnetic particle, 100 μm in diameter, submerged in water at room temperature revolves under the influence of a rotating magnetic field of four permanent magnets of type-N52 Neodymium [26]. The overall density of the spherical particle is assumed to be balanced out by the magnetic pull in the direction of gravitational pull; therefore, it is assumed to be just beneath the surface avoiding sedimentation at the bottom, by design. The rotation rate and the displacement of the magnetic field are controlled by a prismatic-prismatic-revolute joint robotic arm (please see Table 1) articulated by three dedicated DC motors at the respective joints. The spherical magnetic particle is rotated and dragged by the external magnetic field, which generates the vortex in question to help manipulate the selected bacterium, i.e., an *E. coli* minicell [25]. The magnets are located at the edges of a square of the same length. The revolute joint, i.e., the said arrangement of magnets, and the micro-tweezers, i.e., the spherical magnetic particle, are perfectly aligned at *t* = 0 s. The alignment is broken once the arm starts following the control signal and the vortex core traverses along XY-plane (see Figure 2). The robotic arm is expected to stay ahead, whereas the vortex is expected to lag. Furthermore, the *E. coli* minicell is assumed to be unmodified and possessing no magnetic properties, and thus, it is not going to be directly affected by the magnetic fields as magneto-tactic bacteria, such as Magnetospirillum *Gryphiswaldense* [27] or a modified *E. coli* [28], reported in the literature, would have reacted.

Figure 2 depicts the hydrodynamic tweezers moving along the XY-plane as the *E. coli* minicell orbiting its core. The resultant orbit is a transient gait that evolves along with the rigid-body motion of the vortex core. As the magnetic particle moves under the influence of the external magnetic field, the bacterium experiences a transition from the orbit at the instant “*t*” to the new orbit at the instant of “*t* + Δ*t*”. This is only possible if the bacterium is trapped by the forced vortex: the induced pressure difference of the circulation pushes the bacterium in the negative radial direction, i.e., towards the vortex core. Meanwhile, the circulating flow field drags the bacterium in a curvilinear path. The bacterium can propel itself in any random direction, but the thrust force of the rotating tail is not comparable enough to escape from this trap. In return, the bacterium is forced to follow the vortex core as the invoked rigid-body rotation of the spherical magnetic particle drives the flow and pressure fields, while inertial forces do not dominate the overall dynamics for the bacterium cell. The two-way coupled equations of motion for the bacterium cell and the vortex core govern the overall interaction between the two while predicting the velocity field with the associated pressure and drag force profiles. The following detailed model further explains how this interaction occurs.

The rigid-body motion of the bacterium is a cumulative result of propulsion and drag. It is the rotation of the helical tail and body in opposite directions along with the simultaneous hydrodynamic and the tactile interactions with the forced vortex that govern the acceleration. The interaction between the bacterium and the spherical magnetic particle at the center is somewhat complex. The phenomenon could be explained by studying the elements separately by assuming Stokesian flow dynamics and elastic collisions [14,29]; however, the problem is complicated given that all of these effects are inevitably coupled as all depend on the instantaneous position of the bacterium with respect to the spherical magnetic particle.

The equation of motion (1) for the bacterium is given as follows:

(1) $$\left[\begin{array}{cc} m_{b} & 0\\ 0 & j_{b} \end{array}\right]\left[\begin{array}{c} \frac{dU_{b}}{dt}\\ \frac{d^{2}\Theta_{b}}{dt^{2}} \end{array}\right]=\left[\begin{array}{c} F_{p}+F_{d}+F_{v}+F_{c}+F_{\mathrm{lub}+\mathrm{fd}}+F_{\mathrm{add}}\\ T_{p}+T_{d}+T_{\mathrm{lub}+\mathrm{fd}}+R_{b\to v}T_{s} \end{array}\right]$$

governing the six-degrees-of-freedom (6-dof) motion in the inertial frame of the vortex with the rigid-body traverse of **U***_b_* = [*u_r_ u_θ_ u_z_*]^T^ and rigid-body rotation of d**Θ***_b_*/d*t* = [*ω_r_ ω_θ_ ω_z_*]^T^. It should be donated that these two vectors arise due to the circulating flow field only. The core of the matrix moves, and the bacterium realigns itself under the influence of the pressure force and azimuth drag. This behavior is independent of its own propulsive behavior. The propulsive effect of the bacterium tail in the equation above is denoted by **F***_p_*, i.e., the force vector with the subscript “*p*”, respectively. These are calculated based on the resistive-force theory that has been studied extensively [30,31]. It should be noted that the propulsive effect is also dependent on the proximity of the bacterium and the vortex core [32]. The rest of Equation (1) can be listed as follows: (i) the viscous drag vector acting on the bacterium cell due to its own motion following the propulsion of the rotating helical tail, **F***_d_*; (ii) the vector of drag and pressure forces purely induced by the vortex itself [14,29], **F***_v_*; (iii) the contact force when an elastic collision occurs [14,29], **F***_c_*; (iv) the dampening effect due to lubrication and film damping of the fluid film exhibited with proximity [14,29], **F**_lub+fd_; and (v) the effect of added mass on the azimuth direction where the material derivative can be approximated effortlessly [33], **F**_add_. Similarly, the torque components can be defined as (i) the propulsive torque of the rotation tail [30,31], **T***_p_*; (ii) the pure drag of rigid-body rotation, [30,31], **T***_d_*; (iii) the torque due to lubrication and film-damping effects [14,29], **T**_lub+fd_; and (iv) the shear torque on the bacterium body due to asymmetries of the flow field along the radial and vertical directions [14,29], **T***_s_*. Here, **T***_s_* should be projected on the inertial frame of the vortex from the bacterium frame via an associated instantaneous rotation matrix, **R***_b_***_→_***_v_*, for it is originally calculated in the inertial frame of the bacterium cell. The terms on the left-hand side are diagonal mass, **m***_b_*, and moment of inertia, **j***_b_*, matrices associated with the bacterium.

If one is to investigate these components in more depth in the respective order, the equation

(2) $$F_{p}=G_{\mathrm{tail}}\left[\begin{array}{c} 0\\ 0{\hat{e}}_{r}\\ 0{\hat{e}}_{\theta }\\ \omega_{\mathrm{tail}}{\hat{e}}_{z} \end{array}\right]$$

represents the propulsion force with the help of the three-by-six translation-resistance matrix, **G**_tail_, of the rotating helical tail of the bacterium along with its reported rotation rate, *ω*_tail_, harnessing the necessary thrust force [25,30,31]. The **0** on the right-hand side denotes a three-by-one vector with all elements being equal to zero. Also, ${\hat{e}}_{r}$, ${\hat{e}}_{\theta }$, and ${\hat{e}}_{z}$ denote the unit vectors in the inertial frame of the forced vortex, respectively. The ensuing hydrodynamic drag to the overall rigid-body motion of the body of the bacterium cell is given by

(3) $$F_{d}=-\zeta_{r}u_{r}{\hat{e}}_{r}-\zeta_{\theta }u_{\theta }{\hat{e}}_{\theta }-\zeta_{z}u_{z}{\hat{e}}_{z}$$

with $\zeta_{r}\;$, $\zeta_{\theta }$, and *$\zeta_{z}$* denote the effective drag coefficients pertaining radial, azimuth, and perpendicular directions [34,35], respectively.

Next, the force vector comprising radial pressure and azimuthal drag, associated with the presence of a nearby forced vortex as discussed before, is predicted as follows:

(4) $$F_{v}=\left[\begin{array}{c} -\pi a^{2}\rho \int_{r_{b}\left(t\right)-a}^{r_{b}\left(t\right)+a}\frac{{\left(\Omega_{z}\frac{R^{2}}{r}-\omega_{z}\left(r-r_{b}\left(t\right)\right)\right)}^{2}}{r}dr{\hat{e}}_{r}\\ \zeta_{\theta }\Omega_{z}\frac{R^{2}}{r_{b}\left(t\right)}{\hat{e}}_{\theta }\\ 0{\hat{e}}_{z} \end{array}\right].$$

Here, in Equation (4), *a* denotes the radius of the body of the *E. coli* Minicell [25]; *ρ* denotes the density of the liquid medium, i.e., water at room temperature; *r_b_*(*t*) is the radial distance of the bacterium cell to the vortex center; *Ω_z_* is the rotation rate of the spherical magnetic particle; *R* is the radius of the spherical magnetic particle; and *r* stands for the radial position in the vortex frame over which the integral is taken. Here, it is assumed that there is no considerable force along the z-direction; thus, zero denotes no contribution in the respective direction. This vector equation yields negative pressure force regardless of the direction of the vortex rotation, thereby pushing the trapped particle always in the negative radial direction. On the other hand, the azimuth component is observed to be in favor of the direction of rotation. Once the vortex core is incrementally displaced, these force components will be affected but in magnitude alone. The trapped particle will again be compelled to follow a similar path. It can be argued that the rest of the interaction effects arise secondary to the **F***_v_* vector as described here.

The contact force is given with a simple condition in part on the direction of motion expressed as follows:

(5) $$F_{c}=\left(-c\delta_{r}-u_{r}b\left(u_{r}<0\right)\right){\hat{e}}_{r}$$

with *c*, *δ_r_*, and *b* being the stiffness coefficient, penetration depth, and damping coefficient at the point of contact with the magnetic particle [14,29], respectively. Surfaces can further be tailored to repel cells [36], leading to customized contact coefficients. Here, the coefficients are set to simulate repulsion upon tactile contact. But, before the tactile contact occurs, an additional hydrodynamic interaction takes place that can be modeled as follows:

(6) $$F_{\mathrm{lub}+\mathrm{fd}}=\left[\begin{array}{c} -\frac{6\pi \mu a^{2}u_{r}}{h}{\hat{e}}_{r}\\ -6\pi \mu au^{\theta -\mathrm{rel}}\log\left(\frac{h}{a}\right){\hat{e}}_{\theta }\\ 0{\hat{e}}_{z} \end{array}\right],$$

with *μ*, *h*, and $u^{\theta -\mathrm{rel}}$ giving the dynamic viscosity of water at room temperature, the distance along the common normal of the bacterium and spherical magnetic particle surfaces, and the relative azimuth-velocity between the surfaces at proximity [29,37,38] respectively. A lubrication effect is felt along the azimuth, whereas the film damping is felt in the radial direction, prior to contact.

The added mass effect on the bacterium cell is incorporated with the inclusion of the material derivative, D/D*t*, as follows:

(7) $$F_{\mathrm{add}}=\frac{\rho \vartheta_{b}}{2}\frac{Du_{\theta }}{Dt}{\hat{e}}_{\theta },$$

assuming that the main component of the motion in the vortex frame will be along the direction of the azimuth [33]. Here, $\vartheta_{b}$ is the reported volume of the bacterium cell [25].

The torque components in Equation (1) are given similarly as follows:

(8) $$T_{p}=E_{\mathrm{tail}}\left[\begin{array}{c} 0\\ 0{\hat{e}}_{r}\\ 0{\hat{e}}_{\theta }\\ \omega_{\mathrm{tail}}{\hat{e}}_{z} \end{array}\right],$$

with **E**_tail_ being the three-by-six matrix of rotation-resistance in the vortex frame [30].

Furthermore,

(9) $$T_{d}=-\zeta_{rr}\omega_{r}{\hat{e}}_{r}-\zeta_{\theta \theta }\omega_{\theta }{\hat{e}}_{\theta }-\zeta_{zz}\omega_{z}{\hat{e}}_{z}$$

gives the torque acting on the bacterium cell due to rigid-body rotation with $\zeta_{rr}$, $\zeta_{\theta \theta }$, and $\zeta_{zz}$ denoting the effective rotational drag coefficients pertaining radial, azimuth, and perpendicular directions [34,35], respectively, in the vortex frame akin to Equation (3).

The torque induced by lubrication and film-damping is modeled with the help of a simple cross-product given as follows:

(10) $$T_{\mathrm{lub}+\mathrm{fd}}=\left(a{\hat{e}}_{r}\right)\times \left(F_{\mathrm{lub}+\mathrm{fd}}\right).$$

The final torque component acting on the bacterium arises due to asymmetries in the flow field of the forced vortex and modeled as follows [29]:

(11) $$T_{s}=\left[\begin{array}{c} -sgn\left(z_{b}\left(t\right)\right)\frac{8\Omega_{z}\pi a\mu z_{b}\left(t\right)\left(a^{2}+3{\left(z_{b}\left(t\right)\right)}^{2}\right)}{3r_{b}\left(t\right)}{\hat{e}}_{r}\\ 0{\hat{e}}_{\theta }\\ -2\Omega_{z}\pi R^{2}\left(2a-r_{b}\left(t\right)\log\left(a+r_{b}\left(t\right)\right)+r_{b}\left(t\right)\log\left(r_{b}\left(t\right)-a\right)\right){\hat{e}}_{z} \end{array}\right].$$

Here, *z_b_*(*t*) is the instantaneous position of the bacterium along the vertical axis [29]. The said asymmetries arise due to unequal radial and vertical distance between arbitrary points on the spherical surfaces of the magnetic particle and the bacterium body that result in additional rotation in return.

Once the equation of motion and respective frames are established for the bacterium cell, the next step is to articulate the equation of motion for the spherical magnetic particle in the same fluid medium. The most important stimuli to account for are the magnetic field and the drag of the experienced relative flow field due to its own rigid-body motion. The other forces exerted on the core of the vortex can be omitted based on a sole inertia comparison in between the spherical magnetic particle and the bacterium cell. However, lubrication force is included given the low-Reynolds-number flow condition. Therefore, the equation of motion preferred in this study is given as follows:

(12) $$\left[\begin{array}{cc} m_{\mathrm{mag}} & 0\\ 0 & j_{\mathrm{mag}} \end{array}\right]\left[\begin{array}{c} \frac{dU_{\mathrm{mag}}}{dt}\\ \frac{d\Omega_{\mathrm{mag}}}{dt} \end{array}\right]=\left[\begin{array}{c} F_{m}+F_{d-\mathrm{mag}}-R_{b\to v}F_{\mathrm{lub}}\\ T_{m}+T_{d-\mathrm{mag}}-\left(R{\hat{e}}_{r}\right)\times R_{b\to v}F_{\mathrm{lub}} \end{array}\right]$$

for 6-dof motion in the inertial frame of the vortex due to the rate of rigid-body traverse, i.e., **U**_mag_ = [*v _x_ v _y_ v _z_*]^T^, and the rate of rigid-body rotation, i.e., **Ω**_mag_ = [*Ω _x_ Ω _y_ Ω _z_*]^T^, [14,29]. It should be noted that additional interaction effects shall be included should the size and inertia of the two particles become comparable.

The magnetic force vector acting on the vortex core is given as follows:

(13) $$F_{m}=\left(M_{p}\vartheta_{\mathrm{mag}}\right)\cdot \left(R_{r\to v}\nabla B\right),$$

with rotation matrix, **R***_r_*_→*v*_, to project the gradient of the magnetic field, **B**, from the frame of the robotic arm to the frame of the forced vortex. Here, $\vartheta_{\mathrm{mag}}$, is the volume of the spherical magnetic particle. The term **M***_p_* is the instantaneous total magnetization vector of the particle in question and allows one to calculate the magnetic stimuli at any given instant, in the following form:

(14) $$M_{p}=\gamma mL\left(\frac{R_{r\to v}Bm}{kT}\right)$$

with *T*, *k*, *m*, and *γ* denote the absolute temperature of the fluid, Boltzmann constant, magnetization value for a single super paramagnetic iron oxide nano particle, and number density of the particles in the spherical magnetic object [39,40,41,42], respectively. Here, the number density is found via the mass fraction of the nano particles mixed with resin along with the neutral buoyancy assumption, assuming the bulk resin density is around 700 kg/m^3^. Also, L is the Langevin function [41] representing the magnetization profile of the nano particles. The magnetic field is in fact calculated as the linearly superimposed magnetic fields of four separate permanent magnets placed in a square formation, i.e., **B** = [Σ*B_x_* Σ*B_y_* Σ*B_z_*]^T^, expressed in the frame of the end-effector of the robotic arm [43] yielding symmetry along the *Z*-axis of the respective degrees of freedom. Furthermore, each magnetic field component is expressed as a combination of three intricate spatial functions, G_(1,2,3)_, so that the field vector and its gradient can be properly calculated [44]:

(15) $$B_{\left(x,y,z\right)}=\frac{\mu_{0}{Μ}_{\mathrm{mag}}^{\mathrm{robot}}}{4\pi }G_{\left(1,2,3\right)}\left(x^{\mathrm{rel}},y^{\mathrm{rel}},z^{\mathrm{rel}}\right),$$

where ${Μ}_{\mathrm{mag}}^{\mathrm{robot}}$ is the magnetization of the selected material, i.e., type-N52 Neodymium [26] for this study. Furthermore, *μ*_0_ denotes the permeability of empty space, while *x*^rel^, *y*^rel^, and *z*^rel^ signifing the relative position of the magnetic spherical particle with respect to each magnet, respectively.

The fluid drag on the spherical magnetic particle is given by the following:

(16) $$F_{d-\mathrm{mag}}=-6\pi \mu RU_{\mathrm{mag}}.$$

The torque components in Equation (12) are also presented by the following:

(17) $$T_{m}=\vartheta_{\mathrm{mag}}M_{p}\times \left(R_{r\to v}B\right)$$

and

(18) $$T_{d-\mathrm{mag}}=-8\pi \mu R^{3}\Omega_{z}{\hat{e}}_{z}.$$

Equations (1)–(18) are used for the deterministic prediction of rigid-body accelerations. However, it is known that there will be stochastic displacement of the bacterium cell due to Brownian noise [35]. A simple approach to include such an effect is arguably to superimpose this random motion and the deterministic calculations of rigid body motion, i.e., **d***_b_*(*t*) = [*r_b_* (*t*) *r_b_* (*t*) *θ_b_* (*t*) *z_b_* (*t*)]^T^ and **Θ***_b_*(*t*) = [*θ*_X_ (*t*) *θ*_Y_ (*t*) *θ*_Z_ (*t*)]^T^, as follows [35]:

(19) $$r_{\Sigma }\left(t+\delta t\right)=d_{b}\left(t+\delta t\right)+\Gamma \left(\delta t\right),$$

(20) $$\Gamma \left(\delta t\right)={\left[\psi_{r}{\left(2\frac{kT}{\zeta_{r}}\delta t\right)}^{0.5}\psi_{\theta }{\left(2\frac{kT}{\zeta_{\theta }}\delta t\right)}^{0.5}\psi_{z}{\left(2\frac{kT}{\zeta_{z}}\delta t\right)}^{0.5}\right]}^{T},$$

(21) $$\Psi_{\Sigma }\left(t+\delta t\right)=\Theta_{b}\left(t+\delta t\right)+\Phi \left(\delta t\right),$$

and

(22) $$\Phi \left(\delta t\right)={\left[\psi_{rr}{\left(2\frac{kT}{\zeta_{rr}}\delta t\right)}^{0.5}\psi_{\theta \theta }{\left(2\frac{kT}{\zeta_{\theta \theta }}\delta t\right)}^{0.5}\psi_{zz}{\left(2\frac{kT}{\zeta_{zz}}\delta t\right)}^{0.5}\right]}^{T}.$$

Here, each separate term ψ_{*r*, *θ*, *z*, *rr*, *θθ*, zz}_ used in Equations (20) and (22) denotes a random selection of ±1 with uniform distribution over time for the respective degree-of-freedom such that the jumps will take place in random directions along all axes. Moreover, δ*t* stands for the duration of the spatial and rotational jumps, i.e., the time-steps taken by the ODE solver. Therefore, the resultant position and orientation of the bacterium cell at the end of each time step, i.e., r_∑_(*t* + δ*t*) and Ψ_∑_(*t* + δ*t*), are employed in the equations of motion for the calculation of the next iteration. It is important to acknowledge that Ψ_∑_(*t* + δ*t*) is presented in terms of Euler angles [43].

Finally, we have the equations for the robotic arm of three-degrees-of-freedom with the Denavit–Hartenberg parameters provided by Table 1. The selection of the parameters drastically simplifies the inverse kinematics [43]. Each degree of freedom is articulated by its own dedicated DC-motor of the same type, i.e., EC 45 Flat brushless 48 V & 70 W (Maxon Group). The respective equations representing the dynamics of the robotic arm and the DC motors at the joints are as follows:

In Table 1, the twist angles, *α*_{tower,1,2,3}_, represent the x-rotation of each link with respect to the previous joint; the lengths *l*_{tower,1,2,3}_ represent the distance between two consecutive joints residing on the same link and along the *X*-axis associated with the latter; offsets *d*_{tower,1,2,3}_ represent the distance between two consecutive joints residing on the same link and along the *Z*-axis associated with the latter; and the rotations *θ*_{tower,1,2,3}_ denote the rotation between two consecutive joints residing on the same link and along the *Z*-axis associated with the latter. Now, it should be acknowledged that, at the end of the third link, there are four permanent magnets constituting the end-effector with no additional geometric features to account for. Also, the tower in Table 1 serves as an initial offset to initially position the magnets over the fluidic medium, only.

(23) $$D_{\mathrm{arm}}\frac{d^{2}q_{\mathrm{arm}}}{dt^{2}}=K_{\mathrm{motor}}I_{\mathrm{motor}}.$$

Above, in Equation (23), the terms **D**_arm_, **q**_arm_, **K**_motor_, and **I**_motor_ give the effective diagonal inertia matrix for the robotic arm owing to the prismatic–prismatic–revolute joint arrangement as presented in Figure 1 and Table 1, the generalized coordinates for the robotic arm, i.e., the open-kinematic chain as **q**_arm_ = [X Y *θ*_Z_ ]^T^, the diagonal torque constant matrix, and the motor current vector, respectively [43]. Here, *θ*_Z_ denotes the rigid-body rotation of the third link with the permanent magnets depicted in Figure 1. Also, we use the following equation of

(24) $$L_{\mathrm{motor}}\frac{dI_{\mathrm{motor}}}{dt}+R_{\mathrm{motor}}I_{\mathrm{motor}}=V_{\mathrm{motor}}-K_{\mathrm{emf}}\frac{dq_{\mathrm{robot}}}{dt},$$

with **L**_motor_, **R**_motor_, **V**_motor_, and **K**_emf_ signifying the diagonal inductance matrix, armature current vector, diagonal resistance matrix, motor voltage vector, and diagonal friction coefficient matrix, respectively. This last matrix equation brings all the electromechanical properties associated with the three DC motors used at the joints together [43].

Finally, having the entire dynamics expressed in this form leads to the control law summoned in this study. As discussed before, once a position reference is provided, the end-effector is expected to traverse the magnetic field and the micro-tweezers are expected to follow with a variable reaction time, mostly owing to the viscous drag accompanied by the magnetic response of the super paramagnetic nanoparticles. Therefore, a novel approach was investigated with a weighted control signal of two distinct tracking errors: both being used for proportional and integral (PI) control. Furthermore, the integral control is of an adaptive nature [45,46] to tune the output according to the error. The control law and the control signal, *η*, pertinent to the X- and Y-axes, i.e., the laboratory frame, used for this study are as follows:

(25) $$\eta_{\left(X,Y\right)}=\beta \left(\int_{-\infty }^{\tau }\frac{1}{{\left(f_{R-\left(X,Y\right)}k_{i}\right)}^{2}+1}dt+f_{V-\left(X,Y\right)}k_{p}\right)+\left(1-\beta \right)\left(\int_{-\infty }^{\tau }\frac{1}{{\left(f_{V-\left(X,Y\right)}k_{i}\right)}^{2}+1}dt+f_{R-\left(X,Y\right)}k_{p}\right),$$

with *f*_R_ and *f*_V_ indicating the tracking error for the end-effector of the robot and the spherical magnetic particle, respectively. Therefore, the controller is incorporating two distinct velocity and position informations. The proportional and integral gains are denoted by *k_p_* and *k_i_*, respectively. The formulation of the integral control is of an adaptive nature, whereas the proportional gain is constant [43,45,46]. It is also noted that only one set of PI-control gains is used for these two degrees of freedom. The weighing coefficient *β* ensures that the total contribution adds up to unity. The role of the integral gain is to minimize the steady-state error but here, unless *β* ≈ 0.5, the control law will either focus on the end-effector or on the vortex core to achieve the best performance. Since there is no tactile contact or tether between the third degree-of-freedom and the spherical magnetic particle, this approach will yield the best performance for one at the expense of the performance of the other. Furthermore, it should be argued that there are three criteria that directly affect the determination of *k_i_*, *k_p_*, and *β*: (i) the tracking error that is being minimized, (ii) the reaction time of the overall system, and (iii) the level of misalignment between the vortex core and the third degree-of-freedom. Here, it can be argued that the last criterion is favored at the expense of others.

As opposed to the XY-traverse, the control signal generated for the third degree-of-freedom, i.e., the rotation rate of the magnets, is a simpler P and adaptive-I control as *η_z_* = *k_p_f*_R*−Ω*_ + ∫ 1/((*k_i_ f*_R*−Ω*_)^2^ + 1) d*t*, with *f*_R-*Ω*_ representing the error between the rotational velocity reference. Here, the calculated error is solely based on the rotation rate of the third degree-of-freedom. It should be noted that detecting the rotation rate of the vortex core might be impractical in real applications; therefore, it is not included in the control law. It should be noted that there are three control inputs but twelve degrees-of-freedom in total, i.e., the first three belonging to the robotic arm, then three more associated with the spherical magnetic particle, and finally six degrees-of-freedom for the untethered swimming of the bacterium cell. Therefore, the model here represents an under-actuated multi-scale robotic system.

## 3. Results

**Figure A1:**
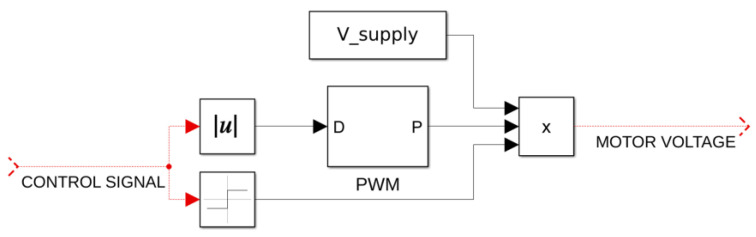
SIMULINK schematic to generate PWM signal for a given control signal to drive the associated DC motor.

**Figure A2:**
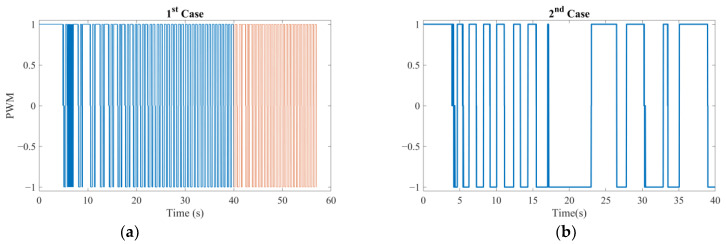
PWM signals on the third degree-of-freedom for the control tasks in this study: (**a**) PWM signals before (in blue) and after (in red) the direction of motion was altered; (**b**) PWM signal while dragging the vortex core with a relatively greater velocity reference.

The following is a detailed numerical study of two separate motion control cases to demonstrate the simulated dynamics of the multi-scale system described thus far: (i) discrete velocity reference to observe the dynamic response to a change in direction of the reference and (ii) dragging the vortex with a high-velocity reference. The spherical magnetic core of the vortex is assumed to be 50 μm in radius and it marks the origin point, i.e., the inertial frame, for the bacterium while traversing together in the laboratory frame. Each type-N52 Neodymium magnet located at the tip of the robotic arm is 2 cm × 1 cm × 1 cm in dimensions, and their bottom surface is placed 1 mm above the fluid surface. It should be noted that the supply voltage and the maximum rotation rate of the selected DC motor are always limited, in accordance with the data sheet supplied by the manufacturer [47]. The control signal is used to generate the necessary pulse-width-modulation (PWM) signal with correct voltage amplification to attain the nominal voltage supply determined by the manufacturer, i.e., 48 V. Please see Figure A1 for the PWM signal generation in the SIMULINK environment and Figure A2a,b for PWM signals without amplification.

The velocity reference to the rotation rate of the magnetic field induced by the four permanent magnets in the degrees-of-freedom is set to be 50 rad/s for both cases, regardless of the step-out phenomena this condition leads to. In addition, the control law coefficients *β*, *k_p_*, and *k_i_* in Equation (25) are set to be 0.05, 1.1, and 0.5, respectively, in both of these cases to establish a benchmark. The adaptive integral used for the XY-traverse was observed to improve the control performance with these settings; however, determination of the coefficients proved to be laborious as a systematic but manual tuning was employed. Once again, the alignment between the third degree-of-freedom and the vortex core was the of utmost importance. It should also be noted that, albeit without inclusion of the motion control or the planar traverse of the vortex core, orbital characterization of the hydrodynamic micro-tweezers was carried out elsewhere [14,29]. Therefore, the focus here was deliberately on the coupled dynamic behavior with relatively higher velocities and, arguably, with extreme conditions.

Numerical solutions are obtained in the MathWorks^®^ MATLAB/SIMULINK environment with an auto-selected variable-step ODE solver (MATLAB Version: 24.1.0.2653294 (R2024a) Update 5 & SIMULINK Version: 24.1) performing on a 64-bit Ubuntu Linux (Version: 20.04.6 LTS) laptop computer running on a 12th Gen Intel^®^ Core^TM^ i5-1235U CPU and 16 GB RAM. Each motion control simulation, presented in blue, carried out computations for a 40 s simulation time, which took around 100 s to complete in rapid accelerator mode. However, once the direction of the motion is changed, presented in red, the simulation is cut short with a 20 s simulation-time that lasted less than a minute to complete with the rapid accelerator mode.

### 3.1. Dynamic Response to Sharp Change in Direction of Velocity Reference

**Figure 3:**
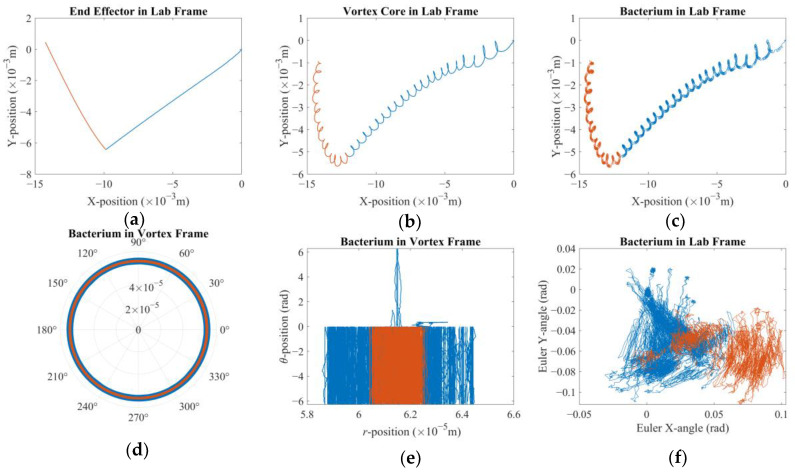
Reaction to a sudden change in direction of velocity reference before and after the change in direction occurs, in blue and in red, respectively: (**a**) position of the tip of the robotic arm; (**b**) position of the vortex core; (**c**) position of the bacterium cell; (**d**) position and orientation of the bacterium cell in the vortex frame in polar coordinates; (**e**) position vs. orientation of the bacterium cell in the vortex frame; (**f**) the orientation of the bacterium cell on the XY-plane given by respective Euler angles.

**Figure 4:**
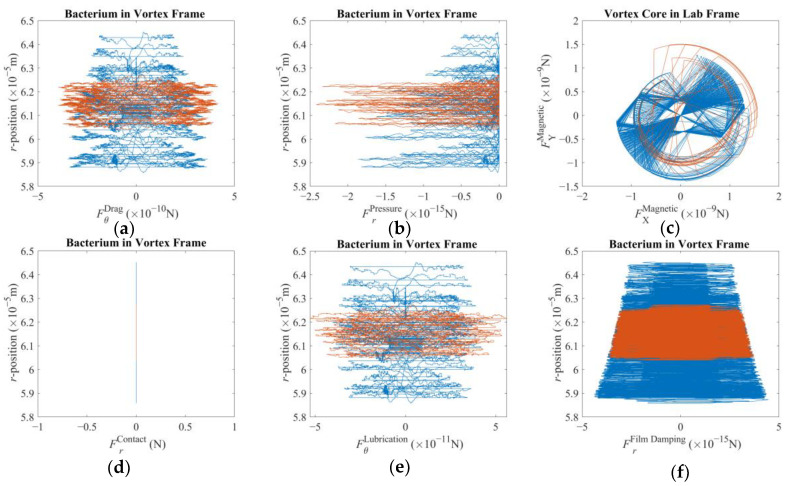
Reaction to a sudden change in direction of the velocity reference before and after the change in direction occurs, in blue and in red, respectively: (**a**) azimuthal drag force on the bacterium; (**b**) radial pressure force on the bacterium; (**c**) magnetic force on the vortex core; (**d**) contact force on the bacterium; (**e**) lubrication force on the bacterium; (**f**) film damping force on the bacterium.

**Figure 5:**
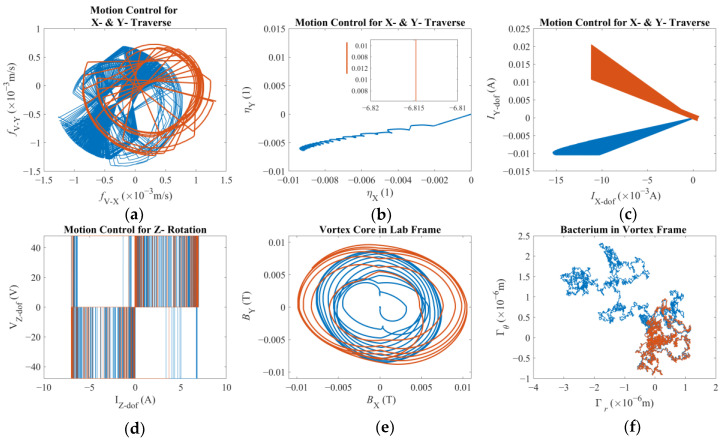
Reaction to a sudden change in direction of velocity reference before and after the change in direction occurs, in blue and in red, respectively: (**a**) tracking error calculated for the vortex core on the XY-plane; (**b**) computed control signal comparison for traverse on the XY-plane with an inset for the second velocity reference (in red); (**c**) motor currents for traverse on the XY-plane; (**d**) amplified PWM signal and motor current for the third degree-of-freedom; (**e**) magnetic field density felt by the vortex; (**f**) Brownian noise for the bacterium.

In the first case, two distinct reference values are applied consecutively to investigate how fast and how accurately the system will react to the change in the direction of the velocity reference. The velocity reference imposed was [−30 × 10^−5^ −30 × 10^−5^ 0]^T^ (m/s) for the first 40 s followed by [0 30 × 10^−5^ 0]^T^ (m/s) for the next 20 s. It is observed that the reaction of the system was much faster than that of the former reference, and 20 s was seemingly enough for the vortex core to travel back along the *Y*-axis; therefore, the simulation was terminated before proceeding further in time. Furthermore, the highest expected Reynolds number of the vortex core is around Re = *ρU2R*/*μ* ≈ 0.038, with *U* being the amplitude of the linear velocity vector, i.e., *U* = |**U**_mag_|. This is well suited for a low-Reynolds-number flow assumption [34,35]. It is also acknowledged that, with low-Reynolds-number conditions, the kinetic energy of the objects is expected to be absorbed quickly, a condition that supports the choice of a small *β* and thus highlights the crucial role of adaptive integral gain applied on the tracking error of the vortex core. Figure 3, Figure 4 and Figure 5 demonstrate how different elements of the system contribute to the overall performance. Figure 3a shows that as soon as the first reference is applied, the end-effector moved along negative X- and negative Y-directions. Due to the given nature of the controller design, it is observed that the robotic arm still exhibits displacement in both X- and Y-axes, once the velocity reference is switched to the latter, presented in red. However, this proves to be crucial for the vortex core to suffice the new reference, as demonstrated in Figure 3b. It is further observed that the robotic arm can react to the new settings virtually instantly; however, the vortex core clearly exhibited secondary order effects and took some time to change its direction from ─Y to +Y. As it did so, the bacterium was also being towed following the same path, as Figure 3c suggests. It should also be noted at this juncture that the reaction time of the vortex core is observed to further increase with an increasing *β* to the point that the control effort simply failed. This observation strengthens the initial hypothesis that the steady-state error of the coupled system should be regulated by the adaptive integral gain over the tracking error of the vortex core. It is clearer with Figure 3d,e of how the bacterium moved around the vortex core. The bacterium cell was captured by the vortex and forced to orbit its core. However, the total angle covered by the bacterium it is not clear in Figure 3d. Therefore, Figure 3e further demonstrates the existence of, and dominance thereof, the step-out phenomenon, which is a well-documented behavior for micro-robotic applications [48], as expected: the observed direction of the vortex rotation, hence the orbital traverse of the bacterium, is opposite to that of the reference. There is but a limited interval in which the rotation occurred in the positive direction. So, it can be argued that the direction of displacement and the step-out behavior combined resulted in this non-intuitive result. Nevertheless, the bacterium cell could not move further away from the vortex core since it was obviously trapped by the induced rotation although it was sporadically being pulled in opposite directions along the azimuth. Finally, it is presented by Figure 3f that the bacterium cell rotated in the lab frame with infinitesimal and apparently random rotation rates under the combined influence of propulsive effects [49], the drag and rotational Brownian noise.

Figure 4 further explains how the vortex core is struggling to keep up with the external stimuli. According to almost symmetrical plots in Figure 4a,e,f, the drag force, the lubrication force, and the film-damping force, respectively, switched rapidly in accordance with a magnetic field rotating too fast for the fluid drag to allow to react in time. Therefore, the super paramagnetic particles faced a magnetic field of the same magnitude but opposite direction before the vortex core could perfectly align itself. This is direct evidence of the step-out phenomenon. However, since the magnetic torque is strong enough, a vortex was formed to force the bacterium orbit around the vortex core, as already depicted in Figure 3d,e. It is also noted that the maximum calculated magnetic force is an order of magnitude larger than the drag force, which stands out as the largest hydrodynamic force predicted. Figure 4b, on the other hand, tells us that the pressure difference was always towards the center of the vortex regardless of the direction of rotation, which is indeed the key element forcing the bacterium to orbit the vortex core [14,29]. The distribution of the magnetic forces, as depicted in Figure 4c, indicates that the vortex core remained close to the axis of rotation of the third degree-of-freedom of the robotic arm. Also, a noticeable eccentricity presented itself once the direction of the velocity reference was switched from negative to positive along the Y-direction with the sudden demand for a full-stop along the *X*-axis. According to Figure 4d, no contact occurred while either the first or the second velocity reference vector is in effect leading to the conclusion that the inertial swirl of the bacterium was strong enough to keep it sufficiently away as it rotated, as already depicted in Figure 3d and Figure 4d.

Figure 5 further reveals details on the dynamics of the coupled system. Figure 5a focuses on the tracking error on the vortex core’s part along the XY-plane. The error to the first velocity reference is dominant given the respective duration of the simulation and both degrees of freedom being articulated simultaneously, as opposed to the second velocity reference vector. Figure 5b demonstrates the control signals for the respective reference vectors. In the first case, the control signal, in blue, is reflecting the tracking error in both X- and Y-directions. On the other hand, for the control signal for the second reference, after the velocity direction is altered, the main effort was exhibited for the Y-direction, whilst the control signal in X-direction remained constant as evident by the inset, both given in red. Figure 5c demonstrates the corresponding motor currents for the simultaneous X- and Y-traverse, whereas Figure 5d represents the amplified PWM signal for the Z-rotation, i.e., the third degree-of-freedom. It is important to note that the calculated current values exceed the maximum continuous current limit reported by the manufacturer, however, without 100% duty cycle intervals with a long duration (please see Figure A2a,b); therefore, no numerical limit was imposed. Figure 5e demonstrates the magnetic field density experienced by the vortex core with X- and Y-directions in comparison. The said values mostly remain below the order of *O*(−2) Tesla suggesting that the spherical magnetic particle remains close to the axis of rotation of the third degree-of-freedom, as already suggested by Figure 4c. Finally, Figure 5f showcases the Brownian jumps exhibited by the bacterium cell along the XY-plane, the order of magnitude of which verifies why such an effect is not included for the vortex core. However, taking the apparent asymmetries and random fluctuations observable in Figure 4a,b,e, it is possible to argue that these jumps should not be dismissed without confirming their relative contribution.

### 3.2. Dragging the Vortex with an Excessive Velocity Reference

**Figure 6:**
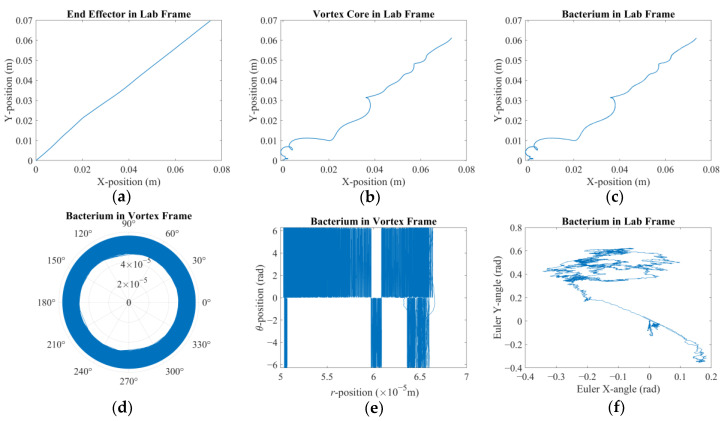
Reaction to constant and comparatively violent pull by the magnetic field: (**a**) position of the tip of the robotic arm; (**b**) position of the vortex core; (**c**) position of the bacterium cell; (**d**) position and orientation of the bacterium cell in vortex frame in polar coordinates; (**e**) position vs. orientation of the bacterium cell in vortex frame; (**f**) the orientation of the bacterium cell on XY-plane given by respective Euler angles.

**Figure 7:**
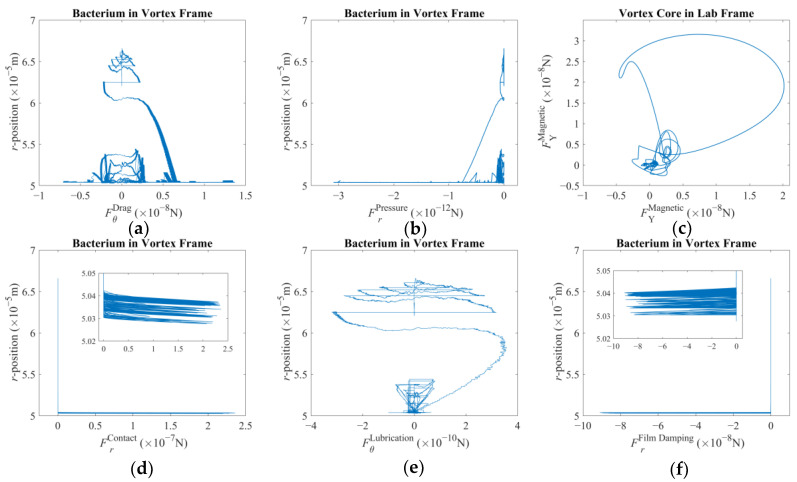
Reaction to constant and comparatively violent pull by the magnetic field: (**a**) azimuthal drag force on the bacterium; (**b**) radial pressure force on the bacterium; (**c**) magnetic force on the vortex core; (**d**) contact force on the bacterium, with the inset further highlighting the non-zero region; (**e**) lubrication force on the bacterium; (**f**) film-damping force on the bacterium, with the inset further highlighting the non-zero region.

**Figure 8:**
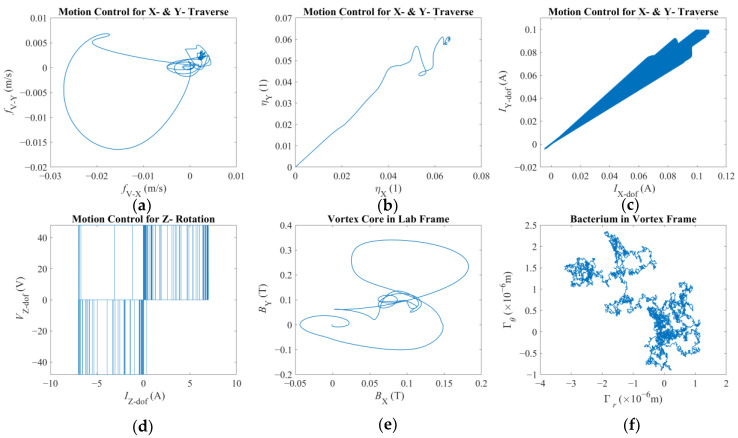
Reaction to constant and comparatively violent pull by the magnetic field: (**a**) error in tracking calculated for the vortex core on the XY-plane; (**b**) computed control signal comparison for traverse on the XY-plane (**c**) motor currents for traverse on the XY-plane; (**d**) amplified PWM signal and motor current for the third degree-of-freedom; (**e**) magnetic field density felt by the vortex; (**f**) Brownian noise on the bacterium.

In the second scenario, a single but drastically faster velocity reference, i.e., [2.5 × 10^−3^ 2.5 × 10^−3^ 0]^T^ (m/s), is imposed to observe the vortex core being dragged in the laboratory frame. This implies that, if the magnetic field is strong enough, all force components are also expected to increase substantially. Furthermore, the Reynolds number of the vortex core is expected to be around Re = *ρU*2*R*/*μ* ≈ 0.32 with *U,* again, being the combined linear velocity. Here, it should be highlighted that the Reynolds number is indicating that inertial forces, such as the added mass given by Equation (7), are becoming relatively more significant. Figure 6, Figure 7 and Figure 8 reveal the reaction of the system with this velocity reference.

Figure 6 depicts the rigid-body behavior for this case: Figure 6a demonstrates that the tip of the robotic arm is following an almost straight path in the XY-plane. Figure 6b,c show that the vortex core was more successful in following the traverse of the magnetic field along the X-direction but fell short along the Y-direction. Figure 6d,e provide further evidence of the previous conjecture: the traverse of the vortex core in the positive direction greatly helped the vortex core and the bacterium cell to rotate in the positive direction, i.e., in the direction of the rotational velocity reference. Therefore, it can be speculated that the displacement direction affects the overall step-out behavior. However, one needs further study to come to a definitive conclusion. It is also observed that the bacterium cell was able to move much closer to the vortex core such that they came into contact. Finally, Figure 6f illustrates the rotation of the bacterium cell in its own inertial frame but expressed in the laboratory frame. It is observed that rotation of the bacterium is an order of magnitude greater than what previously was observed. The overall behavior is expected due to the overall fluid–structure interaction of a bacterium cell [49], but also, it is apparently affected by the conditions of the system modeled here.

Figure 7 presents the force components pertaining to this scenario. The first major difference is the apparent asymmetry in the plots, except the pressure force, which pushes the bacterium towards the vortex core constantly, as expected. The azimuth-drag on the bacterium, as presented in Figure 7a, indicates that the bacterium was pulled in the positive direction as opposed to the previous scenario. This effect is observed to be much stronger when the bacterium was closer to the vortex core. Even when the bacterium is orbiting farther away along the radial direction, the said asymmetry is observable, albeit relatively much weaker. When closer to the vortex core, the drag is two orders of magnitude larger than the previous case, although the difference drops to one order of magnitude with low proximity. This finding agrees with what has been observed in Figure 6e. Pressure force, in Figure 7b, is observed to be three orders of magnitude greater than that of the previous scenario. This, akin to the previous result, is also in agreement with faster orbiting around the vortex core. The magnetic force components felt by the spherical magnetic particle along the XY-plane, as depicted in Figure 7c, are one order of magnitude higher than that of the previous case. This can be explained by the relative position of the vortex core to the magnets with a much faster pace. So, the vortex core was located farther away from the rotation axis of the third degree-of-freedom of the robotic arm. As the distance decreased, a steeper spatial change in the magnetic field was experienced. Figure 7d,f, especially the inset of these said plots, demonstrate that with proximity, the bacterium tends to come into contact with the vortex core. Furthermore, the film-damping effect was acting against separation of the two. It is important to acknowledge that, for a much larger bacterium body, there would be an imbalance and visible wobbling in the rotation of the vortex core. But this effect was not included in the mathematical model; therefore, it does not explain the deviations observed in Figure 6b,c. Next, Figure 7e presents the lubrication force, clearly contributing to the orbital motion of the bacterium, akin to what has been observed in Figure 7a: this effect joins the total effort with as much as one order of magnitude higher push as opposed to the previous case. Furthermore, it is observed that there is an optimum proximity for the lubrication force; it may counteract or contribute but with a relative order of magnitude if the bacterium is too close or far away. This conclusion also required further investigation to determine what affects the optimum radial location. Lastly, lubrication and film-damping forces, given in Figure 7e,f, were predicted to be much greater than those depicted in Figure 4e,f. Especially, the difference between the film-damping forces is on the order of *O*(7), a difference so great that it seems as if there was no film-damping force for orbits with larger radii.

Figure 8 illustrates additional dynamics of this multi-scale system motivated with such excessive velocity reference. Figure 8a depicts that the vortex core was unable to follow the reference such that the error became one order of magnitude larger than what was observed in Figure 3a. Combined with Figure 8b, Figure 8a further demonstrates that the control law could not compensate for the error but kept the vortex core close to the axis of rotation of the third degree-of-freedom. The control signal is found to be one order of magnitude larger, and the control effort is equally apparent in both axes. The resultant motor currents given in Figure 8c are also one order of magnitude larger than what was observed in the previous scenario (as in Figure 5c). These results agree with the velocity reference being larger with the same order. The amplified PWM signal and the motor current on the third degree-of-freedom are given by Figure 8d, and it is reminiscent of what was observed in Figure 5d. It should be noted that the motor currents were not limited in the simulation, and, in both cases, the third DC motor required the highest currents. Figure 8e illustrates the magnetic field density felt by the vortex core, and it is not of a symmetric profile as predicted for the first case (as in Figure 5e), indicating that the spherical magnetic particle is not close to the axis of rotation of the third degree-of-freedom experiencing an uneven magnetic force on different axes. Finally, akin to Figure 5f, Figure 8f illustrates the Brownian jumps the bacterium cell was subject to, which are almost identical in range and rigid-body displacement, as the underlying physical stimuli are assumed to be the same as before, a condition that makes it possible to compare the two cases with higher confidence.

## 4. Discussion

There are two results of this numerical investigation that could be of utmost importance for the future robotic studies of a similar kind. First, displacement and error plots clearly depict that the controller was not able to enforce the velocity reference, as the net displacement fell shorter than the expected value. However, the end-effector of the robotic arm and the vortex core were able to follow each other almost in unison, which, for this study, is the most important criterion in the manual search for control parameters. The velocity reference set to put the overall motion control performance to the test; however, the alignment between the end-effector and the vortex core should not deteriorate beyond what was observed. If two errors were not coupled, arguably, the results would have been completely different. If the error from the robotic arm is considered alone, the arm would have kept up with the velocity reference with higher accuracy but regardless of what happened to the spherical magnetic particle. In the opposite case, if only the error from the vortex is used, virtually, there would have been no limit to the traverse of the end-effector, except its own physical limits, because the error will only increase over time. In either case, one cannot guarantee the existence of the controlled forced vortex, as the distance between the axis of rotation of the third degree-of-freedom and the spherical magnetic particle could become too great for addressable manipulation. In such a case, as the vortex core might become virtually lost, the robotic system must home itself, i.e., search for the magnetic particle and reinitialize the rotation, and then look for the particle to tow, i.e., locate the particle and capture it again. If the particle is a single-celled organism, then simply backtracking the steps might not be enough to recover.

Second, the orbital velocity of the bacterium was deduced to be affected by the displacement direction of the vortex core. The bacterium can exhibit much faster or slower orbital velocities than expected, and even opposite in direction, owing to the step-out phenomenon. Although this requires further careful characterization to fully understand, the implication is promising for robotic applications: the directions of the rotation rate and traverse to the magnetic field would couple either to amplify or attenuate the apparent instantaneous strength of the forced vortex. The direction of the orbital motion might not be of consequence; however, this presented important future research to further ascertain the underlying physics. For instance, the fluid medium might be in motion, e.g., an upstream with a random profile might be present, which would result in a similar behavior. In either case, further numerical study is needed to confirm or dismiss this initial conclusion with a detailed parametric sweep of the relative velocity profile.

It is important to note that such micro-tweezer systems might arguably not be prone to the Brownian noise given that the magnetic field would dominate the overall motion, dampening the possible stochastic effects present. Nevertheless, the bacterium cell itself is subject to such processes, but this phenomenon is not directly included in the equation of motion because the equation set would have been transformed into stochastic differential equations requiring more sophisticated solvers and higher computational resources [50], for the entire system is coupled given how the force and torque components are intricately coupled. Hence, the approach of superimposing the so-called random jumps on the calculated displacements at the end of each time-step was preferred instead.

In this study, it is assumed that all the sensory information was obtained with no latency and with high resolution. Indeed, the dedicated DC motor assemblies at the joints are normally expected to include proper encoders to supply the position and velocity information required for high-performance feedback control. The main question would be to obtain the position information on the vortex core. Depending on the working conditions, the system might defer to visual-serving via cameras [51], fluorescent imaging [52], or acoustics [53]. Sensory equipment could be mounted on the robotic arm or positioned around the fluid medium based on the optimum noise-to-signal ratio. However, the use of magnetic sensing might arguably be almost completely ineffective due to the strong field emanating from the permanent magnets. Furthermore, multiple methods can be fused to track the vortex core and the towed particle. The size difference might even demand such applications as to pick up and release particles in an addressable and controllable manner.

The coefficients *k_p_* and *k_i_* used in this work were simply tuned for the robotic arm only. After which, the weighing coefficient, *β*, is tuned for the coupled error approach. A more robust and rigorous tuning would call for an optimization study on the steady-state error of the system. Furthermore, the former coefficients could be split into two separate sets, i.e., one set for the robotic arm and one set for the vortex core. This, in turn, would increase the demand for computational time and resources for the said optimization task, but more accurate solutions can be achieved.

Finally, the magnetic spherical particle might experience a net vertical force due to the gravitational pull, buoyancy, and gradient of the magnetic field. Here, in this study, equations are written for full six-degrees of freedom; however, these effects are cancelled out by numerically tuning the mass fraction of the resin–nanoparticle mixture. Lighter and heavier resin materials and different types of magnetic nanoparticles are available. Therefore, the nanoparticle content can be modified. The total number of paramagnetic nanoparticles will affect the overall magnetization behavior. Therefore, depending on the application parameters, such as the density or temperature of the fluid medium and the working distance of the permanent magnets, one should determine the optimum set of total volume, total mass, and overall magnetization of the spherical magnetic particle constituting the vortex core. Hence, this stands as a computationally demanding multi-variable multi-objective function design-optimization problem, which might demand case-specific attention. However, a mathematical model, such as the one presented here in this study, will be needed to carry out the said optimization task.

## 5. Conclusions

A robotic manipulation system is envisioned for trapping and towing micro-scale particles. These particles could be of biological origin and require as little tactile contact as possible. The trapping is handled by a hydrodynamic micro-tweezer system, while the manipulation was tasked to a robotic arm articulating a magnetic field of multiple strong permanent magnets. Low-Reynolds-number physics dominates the rigid-body dynamics of the vortex along with this magnetic field. The references values were carefully selected to obtain translational velocities close to the limits of the low-Reynolds-number flow regime and to force the control law to barely keep up with the tracking error. In return, the control law managed to control the position of the tweezers and the tip of the robotic arm simultaneously but at the expense of global accuracy, i.e., it could not keep up with the desired velocity reference vector. However, it has been numerically demonstrated that it is possible to capture and intelligently tow a micro-scale cargo with an under-actuated system using permanent magnets. It should also be noted that electromagnetic coils with computer-controlled currents, although far complicated in construction and in control, will arguably perform much better as the strength, density, and thus gradients of the field could be tuned in accordance with the position of the micro-tweezers in real-time.

## Figures and Tables

**Table 1:** Denavit–Hartenberg parameters for the 3-dof robotic arm, as depicted in Figure 1.

Link #	Twist (rad)	Length (m)	Offset (m)	Rotation (rad)
Tower	*α*_tower_ = 0	*l*_tower_ = 0	*d*_tower_	*θ*_tower_ = 0
1st	*α*_1_ =−π/2	*l*_1_ = 0	*d*_1_(*t*)	*θ*_1_ = 0
2nd	*α*_2_ = −π/2	*l_2_* = 0	*d*_2_(*t*)	*θ*_2_ = 0
3rd	*α*_3_ = 0	*l_3_* = 0	*d*_3_	*θ*_3_(*t*)

## Data Availability

All simulation data will be available upon request.
